# Supplementary material for: A fault-intrusion-tolerant system and deadline-aware algorithm for scheduling scientific workflow in the cloud
Source: PeerJ Comput Sci. 2021 Nov 2;7:e747. doi: 10.7717/peerj-cs.747 (PMC8576554; doi:10.7717/peerj-cs.747)
Supplement: Supplemental Information 6 [file peerj-cs-07-747-s006.zip › Myproject1.1/docs/org/workflowsim/scheduler/BaseScheduler.html]

BaseScheduler


JavaScript is disabled on your browser.


- Overview
- Package
- Class
- Use
- Tree
- Deprecated
- Index
- Help

- Prev Class
- Next Class

- Frames
- No Frames

- All Classes

- Summary:
- Nested |
- Field |
- Constr |
- Method

- Detail:
- Field |
- Constr |
- Method


org.workflowsim.scheduler

## Class BaseScheduler

- java.lang.Object
- - org.workflowsim.scheduler.BaseScheduler

- All Implemented Interfaces:
  :   SchedulerInterface

  Direct Known Subclasses:
  :   DataAwareScheduler, FCFSScheduler, MaxMinScheduler, MCTScheduler, MinMinScheduler, StaticScheduler

  ---

    

  ```
  public abstract class BaseScheduler
  extends java.lang.Object
  implements SchedulerInterface
  ```

  The base scheduler has implemented the basic features. Every other scheduling method
  should extend from BaseScheduler but should not directly use it.

  Since:
  :   WorkflowSim Toolkit 1.0

- - ### Constructor Summary

    Constructors

    Constructor and Description

    `BaseScheduler()`

    Initialize a BaseScheduler
  - ### Method Summary

    Methods

    | Modifier and Type | Method and Description |
    | --- | --- |
    | `java.util.List` | `getCloudletList()` Gets the job list. |
    | `java.util.List` | `getScheduledList()` Gets the scheduled job list |
    | `java.util.List` | `getVmList()` Gets the vm list |
    | `abstract void` | `run()` The main function |
    | `void` | `setCloudletList(java.util.List list)` Sets the job list. |
    | `void` | `setVmList(java.util.List list)` Sets the vm list |

    - ### Methods inherited from class java.lang.Object

      `clone, equals, finalize, getClass, hashCode, notify, notifyAll, toString, wait, wait, wait`

- - ### Constructor Detail


    - #### BaseScheduler

      ```
      public BaseScheduler()
      ```

      Initialize a BaseScheduler
  - ### Method Detail


    - #### setCloudletList

      ```
      public void setCloudletList(java.util.List list)
      ```

      Sets the job list.

      **Specified by:**
      :   `setCloudletList` in interface `SchedulerInterface`

      Parameters:
      :   `list` -


    - #### setVmList

      ```
      public void setVmList(java.util.List list)
      ```

      Sets the vm list

      **Specified by:**
      :   `setVmList` in interface `SchedulerInterface`

      Parameters:
      :   `list` -


    - #### getCloudletList

      ```
      public java.util.List getCloudletList()
      ```

      Gets the job list.

      **Specified by:**
      :   `getCloudletList` in interface `SchedulerInterface`

      Returns:
      :   the job list


    - #### getVmList

      ```
      public java.util.List getVmList()
      ```

      Gets the vm list

      **Specified by:**
      :   `getVmList` in interface `SchedulerInterface`

      Returns:
      :   the vm list


    - #### run

      ```
      public abstract void run()
                        throws java.lang.Exception
      ```

      The main function

      **Specified by:**
      :   `run` in interface `SchedulerInterface`

      Throws:
      :   `java.lang.Exception`


    - #### getScheduledList

      ```
      public java.util.List getScheduledList()
      ```

      Gets the scheduled job list

      **Specified by:**
      :   `getScheduledList` in interface `SchedulerInterface`

      Returns:
      :   job list


- Overview
- Package
- Class
- Use
- Tree
- Deprecated
- Index
- Help

- Prev Class
- Next Class

- Frames
- No Frames

- All Classes

- Summary:
- Nested |
- Field |
- Constr |
- Method

- Detail:
- Field |
- Constr |
- Method
